# High-performance breathable magnetic core for high-frequency power electronic systems

**DOI:** 10.1016/j.fmre.2024.08.008

**Published:** 2024-09-05

**Authors:** Ming Cheng, Wei Qin, Xinkai Zhu, Zheng Wang, Wei Hua

**Affiliations:** a School of Electrical Engineering, Southeast University, Nanjing 210096, China; b Department of Electric Power Engineering, North China Electric Power University, Baoding 071003, China

**Keywords:** Magnetic components, Magnetic materials, Magductance, Magnetic flux skin effect, Breathable magnetic core

## Abstract

The magnetic components (inductors and transformers) that consist of magnetic cores and windings play an important role in power electronic systems. The development of fast-switching semiconductor devices has led to an increase in the working frequencies of power electronic systems. However, high-frequency magnetic flux causes severe loss and heat generation in magnetic cores. This heat is difficult to dissipate owing to the typical solid structure of magnetic cores. Consequently, the risk of heat-induced damage has increased in power electronic systems. Magnetic materials with low conductivities may reduce loss and temperature rise. However, they increase the volume/weight and cost. To solve this problem, we propose a high-performance breathable magnetic core based on the magnetic flux skin effect with magductance. This can reduce the amount of magnetic materials, volume, and weight and facilitate loss reduction and heat exchange. In addition, it provides a new avenue for the study, design, and optimal production of various electromagnetic devices.

## Graphical abstract

**Figure:**
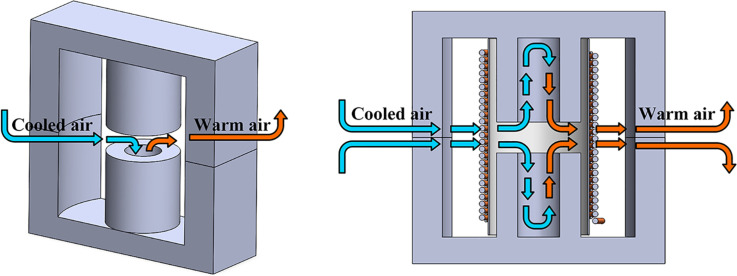

## 1 Introduction

Power electronic systems are crucial for the control and conversion of electrical power. They are widely utilized in power suppliers, power chargers, power conditioners, etc. [[1], [2], [3]]. The advent of fast-switching semiconductor switches, such as the wide bandgap devices fabricated from silicon carbide (SiC) and gallium nitride (GaN), has led to an increase in the working frequencies of power electronic systems. This allows for higher control bandwidths and better power conditioning quality [4]. Magnetic devices, e.g., inductors and transformers, are basic devices in power electronic systems that are used for ripple filtering, energy storage, electric isolation, etc. [5]. Magnetic cores are the carriers of magnetic flux, and they play an important role in determining the performance of magnetic devices [6]. Therefore, magnetic core design is one of the key steps in the design of magnetic components.

The rapid development of power electronic devices with high switching frequencies has reduced the volumes of magnetic cores and thus allowed for compact sizes and low weights of magnetic devices in power electronic systems. However, magnetic cores composed of soft magnetic materials inevitably experience eddy loss and hysteresis loss owing to alternating magnetic flux [5,7]. As the working frequency increases, the iron loss and temperatures in magnetic cores increase at a certain magnetic flux density. Magnetic materials with low conductivities, such as MnZn ferrite materials, are employed in magnetic cores to minimize power loss and temperature rise in devices [8,9]. However, these materials typically have a low saturation magnetic density, resulting in high volume/weight and cost. Consequently, it has become impossible to achieve multiobjective optimization for magnetic cores in terms of compact sizes with low weight, loss, temperature, and cost.

In the conventional design of magnetic components, magnetic cores typically have a solid structure or incorporate an air gap in series with a magnetic circuit [6,10]. This series configuration, where the air gap reluctance is in series with the magnetic core reluctance, can only modify the magnitude of the magnetic flux without affecting its distribution across the cross section of the magnetic core. Magnetic cores wrapped with conductive windings experience eddy current loss, hysteresis loss, and heat generation. As a result, there is weak airflow inside these magnetic cores. This study overcomes the limitations of conventional design and proposes a breathable magnetic core, which allows for the flexible exchange of air inside and outside the core. This breathable magnetic core is based on the discovery of the skin effect in magnetics. A new magnetic component, i.e., magductance [11], is used to derive the distribution of magnetic flux at the cross section of the magnetic core. Magnetic flux is concentrated at the periphery of the cross section of the magnetic core under a high-frequency alternating magnetic field.

Based on the magnetic-flux-concentrating nature of the magnetic flux skin effect, the magnetic core is designed with a bracelet-like shape, where the center of the magnetic core is hollowed. The magnetic core is breathable, which facilitates the exchange of air with a large heat dissipation area. Moreover, the power loss of the proposed magnetic core is reduced owing to the decrease in its magductance under high-frequency field excitation. Consequently, the proposed magnetic core not only reduces the use of magnetic materials and thus volumes/weights but also facilitates loss reduction and heat exchange. The clarification of the mechanism of the magnetic flux skin effect based on magductance provides a new avenue for the study, design, and optimal production of various electromagnetic devices. This innovative discovery will have significant implications for the development of electromagnetic physics.

## 2 Mechanisms and methods

### 2.1 Experimental observation of magnetic flux skin effect

**Fig. 1:**
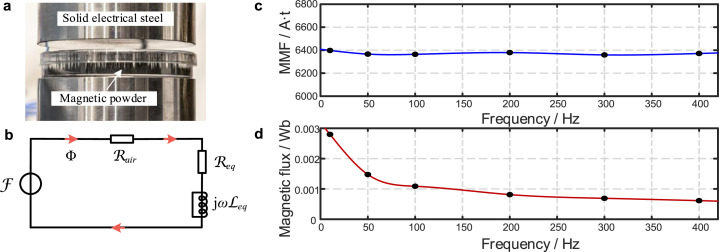
**Observation of magnetic flux skin effect.** (a) Closed magnetic circuit composed of a solid electrical steel and an air gap. The air gap is used to create a layer of magnetic powder for observing the amplitude and distribution of magnetic flux. (b) Equivalent magnetic circuit of the test equipment. F denotes the alternating MMF, and Φ denotes the alternating magnetic flux. R*_air_* denotes the reluctance of the air gap, and R*_eq_* and L*_eq_* denote the equivalent reluctance and equivalent magductance of solid electrical steel, respectively. (c) Magnitude of the MMF remains constant as the test frequency varies. (d) Amplitude of the alternating magnetic flux versus the test frequency.

In a magnetic circuit, an Fe_3_O_4_ magnetic powder is typically used to depict the location and direction of magnetic flux [12,13]. Therefore, we utilize the magnetic powder to demonstrate the magnetic flux skin effect and investigate its cause. In the closed magnetic circuit composed of solid DT4C electrical steel, an air gap is introduced to apply a thin layer of the uniformly distributed magnetic powder, as shown in Fig. 1a. According to the vector magnetic circuit theory [11], the corresponding equivalent magnetic circuit is shown in Fig. 1b. When alternating magnetic flux exists in the magnetic circuit, the magnetic powder is attracted to form a pillar shape. The magnitude and distribution of magnetic flux in the magnetic circuit can be observed according to the height of the pillar shape and the distribution of the magnetic powder. Fig. 1c and d show the test results obtained using a power analyzer while maintaining a constant amplitude of the magnetomotive force (MMF) (6373 A-turn (A·t)) and changing the frequency of the MMF from 10 Hz to 400 Hz. When the MMF is constant, the amplitude of magnetic flux decreases as the frequency of the MMF increases. This implies that equivalent magductance exists in the magnetic circuit. Thus, the accuracy of the equivalent magnetic circuit shown in Fig. 1b is verified. The details of the experiments are provided in Appendix A.

**Fig. 2:**
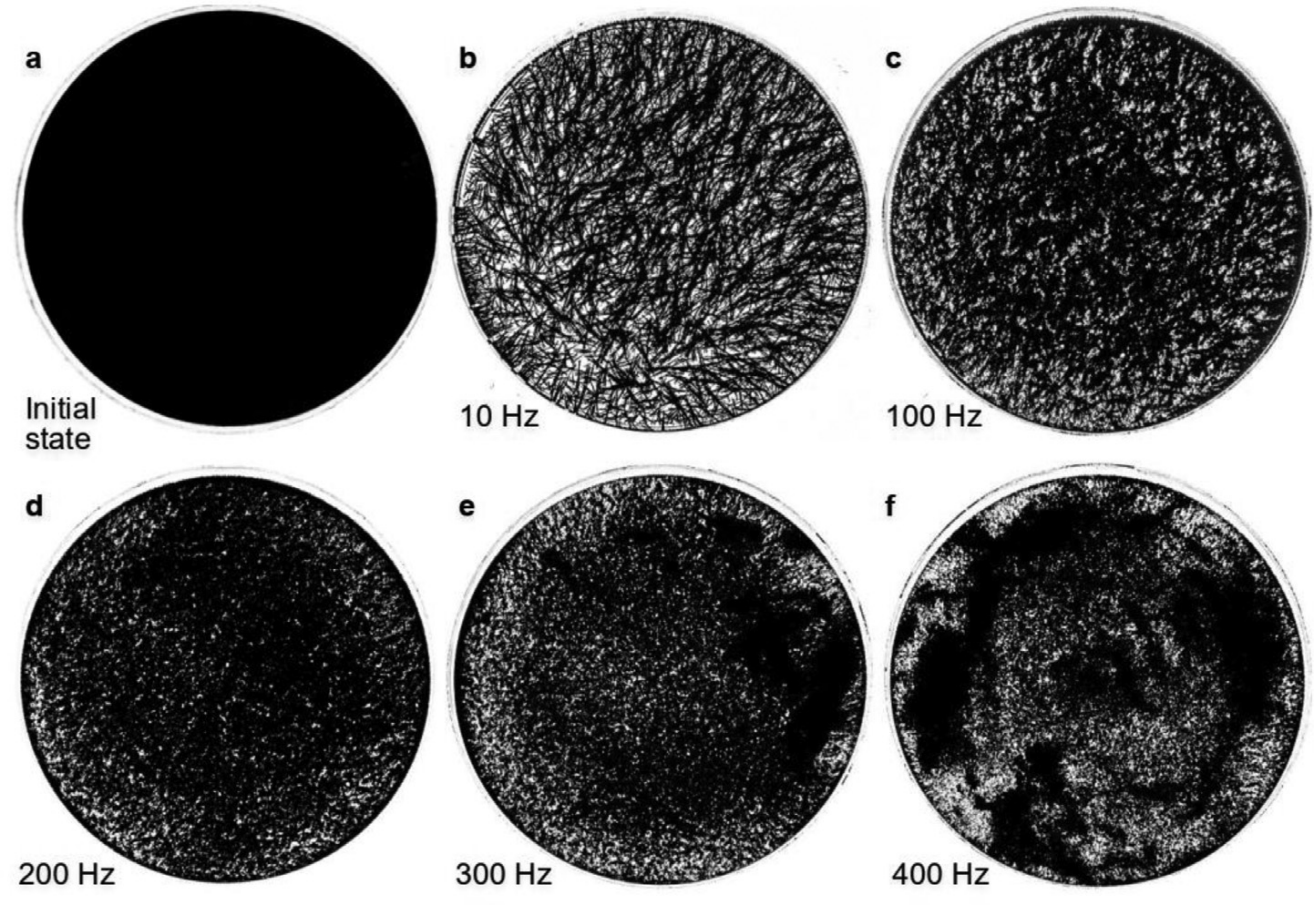
**Distribution of magnetic powder at different frequencies of the MMF.** (a) Uniform distribution of magnetic powder in the case of no alternating magnetic flux in the magnetic circuit. (b) Magnetic powder distribution at a frequency of 10 Hz, indicating that the magnetic powder is attracted to form a pillar shape with uniform distribution under the alternating magnetic flux. (c) Magnetic powder distribution at a frequency of 100 Hz, indicating that the height of pillar shape formed by the magnetic powder decreases, which implies that the amplitude of magnetic flux decreases. The magnetic powder distribution is still approximately uniform. (d) Magnetic powder distribution at a frequency of 200 Hz, indicating that a slight magnetic flux skin effect appears at the center of the magnetic powder. (e) Magnetic powder distribution at a frequency of 300 Hz, indicating the magnetic flux skin effect becomes more evident at the center of the magnetic powder. (f) Magnetic powder distribution at a frequency of 400 Hz, indicating a clear magnetic flux skin effect at the center of the magnetic powder. This proves the existence of magductance in the magnetic circuit.

**Fig. A1:**
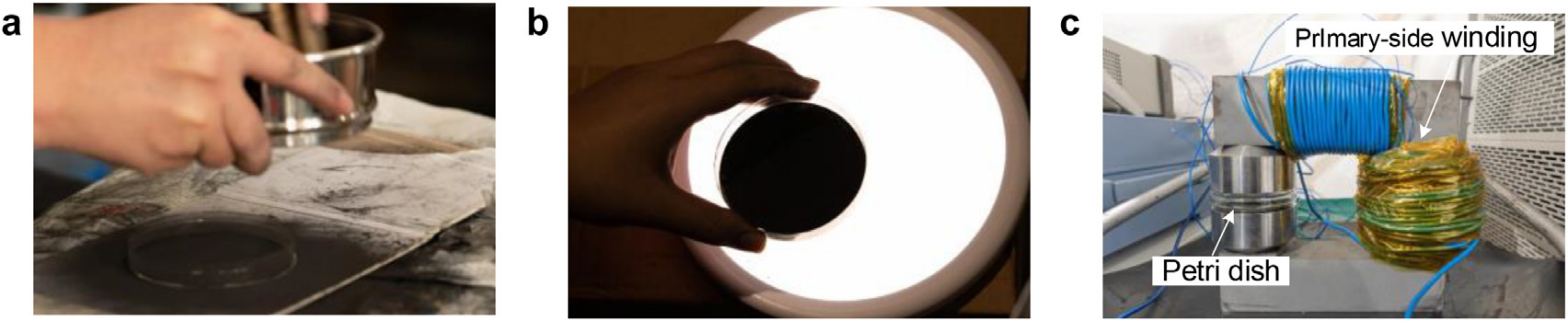
**Procedure of experiment for magnetic flux skin effect.** (a) Magnetic powder is evenly spread on the Petri dish through a sieve. (b) Uniformity of magnetic powder in the Petri dish is checked using a flat lamp. (c) Experimental setup with Petri dish in air gap of electrical steel.

We obtain the corresponding distribution of the magnetic powder at various frequencies of the MMF, as shown in Fig. 2. If there is no magnetic flux in the magnetic circuit, the magnetic powder distribution is uniform (Fig. 2a). Fig. 2b–f show that the pillar length of the magnetic powder decreases as the frequency of the MMF increases, which implies that the amplitude of magnetic flux decreases. These results are consistent with those shown in Fig. 1d. The magnetic flux skin effect is observed using the distribution of the magnetic powder at different frequencies. The magnetic powder close to the center is evenly dispersed at low frequencies. However, the magnetic powder at the center of the container progressively moves to the edge as the frequency increases, and a large blank space forms at the center without the magnetic powder. This is the magnetic flux skin effect. It should be noted that the cross section of the circular Petri dish is larger than that of the tested rectangular magnetic circuit, as illustrated in Fig. A1c in Appendix A. Therefore, the magnetic powder is distributed along the edges of the cross section of a square magnetic circuit at 400 Hz, and partly blank spaces are formed at the edge of the Petri dish.

**Fig. 3:**
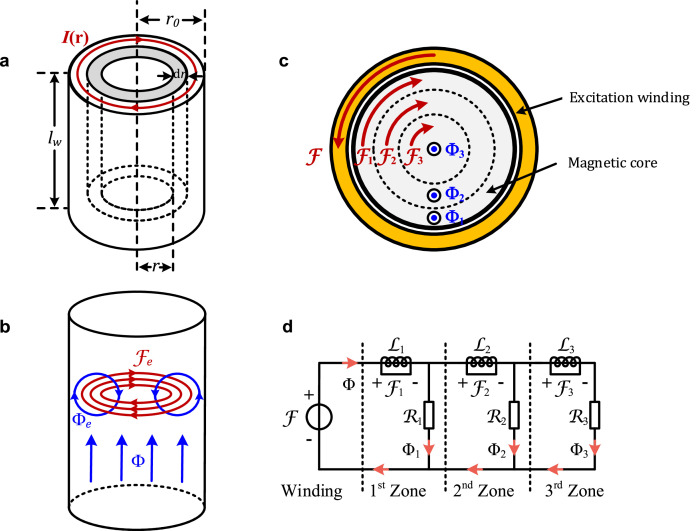
**Magnetic flux skin effect in the cylindrical magnetic core.** (a) Schematic of the cylindrical magnetic core, where *r*_0_ denotes the radius of the cross section, *r* denotes the distance from the center of the cross section, and d*r* is the thickness of a shell in the magnetic core. (b) Schematic of magnetic flux distribution in cylindrical magnetic core, where Φ denotes the alternating magnetic flux, F*_e_* denotes the antimagnetomotive force (eddy current) generated by the alternating magnetic flux, and Φ*_e_* denotes the alternating magnetic flux generated by F*_e_*. (c) Schematic of MMF and magnetic flux distribution in the cross section of a cylindrical magnetic core. F denotes the MMF generated by the excitation winding. F_1_F_2_, and F_3_ denote the MMF caused by eddy-current reaction in the three parts of the magnetic core. Φ_1_, Φ_2_, and Φ_3_ denote the magnetic flux in the three parts of the magnetic core, respectively. (d) Equivalent ladder magnetic circuit of the magnetic core. R_1_, R_2_, and R_3_ denote the reluctance for the three parts of the magnetic core, respectively. L_1_, L_2_, and L_3_ denote the equivalent magductance for the three parts of the magnetic core, respectively.

We remove the cylindrical part of the magnetic core to analyze the cause of the magnetic flux skin effect, and the geometric structure is shown in Fig. 3a. The effects of magnetic flux leakage, magnetic hysteresis, and magnetic saturation are neglected, and alternating magnetic flux circulates within the magnetic core. In accordance with Faraday's law and Lenz's law [6], the accompanying antimagnetomotive force (F*_e_*, eddy current) is generated to prevent the change in magnetic flux (Fig. 3b). Further, the magnetic flux (Φ*_e_*) generated by F*_e_* affects the distribution of the original magnetic flux (Φ) in the magnetic core.

To further analyze the distribution of magnetic flux in the magnetic core, we divide the core into three parts according to the principle of equal cross-sectional areas for simplicity (*S*_1_ = *S*_2_ = *S*_3_, *S*_1_, *S*_2_, and *S*_3_ are the cross-sectional areas corresponding to the three parts of the magnetic core), as shown in Fig. 3c. We assume that magnetic flux is uniform in each part. The influence of magductance on magnetic flux gradually increases with the frequency of magnetic flux. Each part of the magnetic core experiences antimagnetomotive forces (F_1_, F_2_, F_3_), which can be combined with the reluctances (R_1_, R_2_, and R_3_) for each part to determine the magnetic flux (Φ_1_, Φ_2_, and Φ_3_) flowing through each part. We analyze each part of the magnetic core according to Ampere's law [14] and obtain the following:

(1) $$\begin{array}{c} \left\{ \begin{array}{c} F=F_{1}+R_{1}\Phi_{1}\\ F-F_{1}=F_{2}+R_{2}\Phi_{2}\\ F-F_{1}-F_{2}=F_{3}+R_{3}\Phi_{3} \end{array}\right. \end{array}$$

Here, according to the vector magnetic circuit theory [11], considering that the magnetic flux is a sine wave, the magnitude of F*_n_* (*n* = 1, 2, 3) depends on the equivalent magductance L*_n_* (*n* = 1, 2, 3) and magnetic flux Φ*_n_* (*n* = 1, 2, 3) of the magnetic core, that is

(2) $$\begin{array}{c} \left\{ \begin{array}{c} F_{1}=\omega L_{1}\left(\Phi_{1}+\Phi_{2}+\Phi_{3}\right)\\ F_{2}=\omega L_{2}\left(\Phi_{2}+\Phi_{3}\right)\\ F_{3}=\omega L_{3}\Phi_{3} \end{array}\right. \end{array}$$

Combining (1), (2), we obtain

(3) $$\begin{array}{c} \left\{ \begin{array}{c} F=\omega L_{1}\left(\Phi_{1}+\Phi_{2}+\Phi_{3}\right)+R_{1}\Phi_{1}\\ R_{1}\Phi_{1}=\omega L_{2}\left(\Phi_{2}+\Phi_{3}\right)+R_{2}\Phi_{2}\\ R_{2}\Phi_{2}=\omega L_{3}\Phi_{3}+R_{3}\Phi_{3} \end{array}\right. \end{array}$$

The equivalent magnetic circuit corresponding to the magnetic core is shown in Fig. 3d, and we refer to this as a ladder magnetic circuit [15]. In Fig. 3a and b, according to the definition of reluctance [6], we obtain

(4) $$\begin{array}{c} \left\{ \begin{array}{c} R_{n}=\frac{l_{w}}{\mu S_{n}}\\ S_{1}=S_{2}=S_{3} \end{array}\right. \end{array}$$

This yields

(5) $$\begin{array}{c} R_{1}=R_{2}=R_{3} \end{array}$$

Substituting Eq. 5 into Eq. 1 yields

(6) $$\Phi_{1}>\Phi_{2}>\Phi_{3}$$

Consequently, Φ is the largest on the magnetic core's surface and the smallest at its center. This clarifies the mechanism of the magnetic flux skin effect. In addition, when the frequency of magnetic flux in the magnetic circuit is zero or extremely low, the effect of magductance on magnetic flux can be neglected, that is

(7) $$\begin{array}{c} F_{1}=F_{2}=F_{3}=0 \end{array}$$

Substituting Eq. 7 into Eq. 1 yields

(8) $$\begin{array}{c} F=R_{1}\Phi_{1}=R_{2}\Phi_{2}=R_{3}\Phi_{3} \end{array}$$

Therefore, the MMF for the three parts of the magnetic core is the same, the reluctances of the three parts are connected in parallel, and magnetic flux is uniformly distributed. In other words, there is no magnetic flux skin effect. A comparison of (3), (8) shows that the essential cause of the magnetic flux skin effect in the magnetic circuit is the existence of magductance. Therefore, it is important to reduce the influence of magductance on magnetic flux at high frequencies.

### 2.2 General solution for reluctance and magductance of the magnetic core

As the magnetic flux skin effect depends on magductance, it is crucial to accurately calculate magductance. It is assumed that a cylindrical magnetic core with radius *r*_0_ and length *l*_w_ transmits sinusoidal magnetic flux Φ as

(9) $$\Phi =\Phi_{m}\text{cos}\omega t$$

where Φ_m_ is the amplitude of the magnetic flux.

The effects of magnetic flux leakage, magnetic hysteresis, and magnetic saturation are neglected. The one-dimensional magnetic flux distribution in the cylindrical magnetic core is a function of the magnetic core radius (*r*), as shown in Fig. 3a. The magnetic flux density (*B*) and electric field intensity (*E*) inside the magnetic core are described by the modified Bessel functions [16,17].

(10) $$\frac{d^{2}B}{dr^{2}}+\frac{1}{r}\frac{dB}{dr}-j\omega \sigma \mu B=0$$

(11) $$\frac{d^{2}E}{dr^{2}}+\frac{1}{r}\frac{dE}{dr}-\frac{E}{r^{2}}-j\omega \sigma \mu E=0$$

The derivation of the modified Bessel functions for a solid cylindrical conductor is provided in Appendix C. In (10), (11), *ω* denotes the angular frequency of magnetic flux, *σ* denotes the electrical conductivity of the core, *μ* denotes the permeability of the magnetic core, and j denotes an imaginary unit. Solving Eq. 10 yields

(12) $$B\left(r\right)=AI_{0}\left(kr\right)=A\sum_{k=0}^{\infty }{\left(\frac{1}{k!}\right)}^{2}{\left(\frac{kr}{2}\right)}^{2k}$$

where *I*_0_ is the modified Bessel function of the first kind and order zero, *A* is a constant determined by the initial magnetic density of the centroid, and *k* is the complex propagation constant. The square of *k* is given by

(13) $$k^{2}=j\omega \sigma \mu =j\frac{2}{\delta^{2}}$$

where *δ* denotes the skin depth [6,16]. The Bessel function given by Eq. 12 can be expressed by Kelvin functions *ber* and *bei*.

(14) B(r)=A[ber(α)+jbei(α)]

where *α* is given by

(15) $$\alpha =r\sqrt{\omega \sigma \mu }=\frac{\sqrt{2}}{\delta }r$$

Further, we calculate the normalized magnetic flux density distribution and phase relationship based on the magnetic flux density at the surfaces.

(16) $$\frac{B\left(r\right)}{B\left(r_{0}\right)}=\frac{I_{0}\left(kr\right)}{I_{0}\left(kr_{0}\right)}=\left|\frac{B\left(r\right)}{B\left(r_{0}\right)}\right|e^{j\theta_{B}}=\frac{ber(\alpha )+jbei(\alpha )}{ber\left(\alpha_{0}\right)+jbei\left(\alpha_{0}\right)}$$

**Fig. 4:**
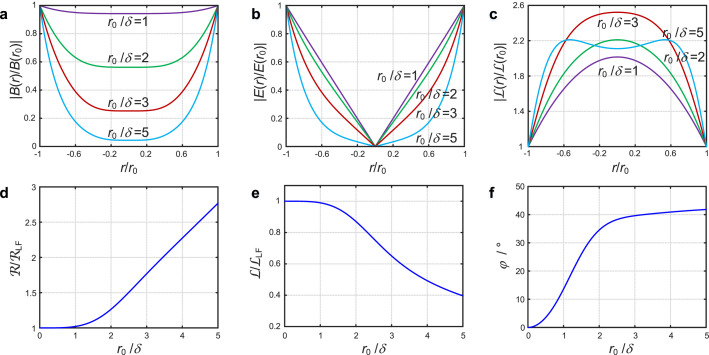
**Analysis of electromagnetic characteristics of the cylindrical magnetic core.** (a) Distribution of normalized magnetic flux density (|*B*(*r)*/*B*(*r*_0_)|) in the cylindrical magnetic core for different skin depths (*δ*). The inhomogeneity of magnetic flux distribution increases with *δ*. (b) Distribution of normalized electric field intensity (|*E*(*r)*/*E*(*r*_0_)|) in the cylindrical magnetic core for different skin depths. The magnetic flux skin effect and eddy current skin effect accompany and affect each other. (c) Distribution of normalized magductance (|L(*r)*/L(*r*_0_)|) in the cylindrical magnetic core for different skin depths. Magductance is the smallest on the magnetic core's surface, and it increases closer to the center of the magnetic core. (d) Normalized reluctance (|R/R_LF_|) increases with *δ*. The equivalent reluctance increases as the equivalent cross-sectional area of the magnetic flux flow decreases. (e) Normalized magductance (|L/L_LF_|) decreases with *δ*. This implies that the eddy current skin effect caused by the magnetic flux skin effect influences magductance. (f) Magnetic impedance angle *φ* in the cylindrical magnetic core for different skin depths. The magnetic impedance angle gradually increases as the skin depth *δ* decreases.

The distribution of magnetic flux theoretically verifies the effect of the magductance in the magnetic core on magnetic flux, as shown in Fig. 4a. In addition, it can mathematically express the magnetic flux skin effect. Solving Eq. 11, the electric field intensity can be calculated as

(17) $$E\left(r\right)=\frac{j\omega \Phi }{2\pi r_{0}}\frac{I_{1}\left(kr\right)}{I_{1}\left(kr_{0}\right)}$$

where *I*_1_ is the modified Bessel function of the first kind and order 1. Similarly, the normalized expression of *E*(*r*) is derived, and the curves are plotted in Fig. 4b.

A comparison of Fig. 4a,b demonstrates the interdependence of the eddy current skin effect and magnetic flux skin effect. Reluctance and magductance are determined by both the effects. In addition, the magnetic flux (Φ) of the circular cross section can be obtained as

(18) $$\begin{array}{ccc} \Phi & = & \int_{0}^{r_{0}}B\left(r\right)2\pi rdr=\frac{2\pi r_{0}A}{k}I_{1}\left(kr_{0}\right)\\ & = & A\pi \sqrt{2}\delta r_{0}\left[bei^{\prime }\left(\alpha_{0}\right)-jber^{\prime }\left(\alpha_{0}\right)\right] \end{array}$$

where *ber*’(*α*_0_) and *bei*’(*α*_0_) are the derivatives of the *ber* and *bei* functions at *α*_0_, respectively. According to the definition of magductance, i.e., the number of moving electric charges generated per unit magnetic flux [18,19], we can obtain the distribution of magductance in the cylindrical magnetic core, as given by Eq. 19. The details are provided in Appendix D.

(19) $$\begin{array}{ccc} L\left(r\right) & = & \frac{\Gamma \left(r\right)}{\Phi \left(r\right)}\\ & = & \frac{l_{w}\sigma }{2\pi }\left\{\frac{1}{kr_{0}}\frac{I_{0}\left(kr_{0}\right)}{I_{1}\left(kr_{0}\right)}-\frac{1}{2}\left(\frac{r}{r_{0}}\right)\left[\frac{I_{0}^{2}\left(\mathrm{kr}\right)}{I_{1}\left(kr_{0}\right)I_{1}\left(\mathrm{kr}\right)}-\frac{I_{1}\left(\mathrm{kr}\right)}{I_{1}\left(kr_{0}\right)}\right]\right\} \end{array}$$

where Γ(*r*) denotes the charge linkage generated by the magnetic flux (Φ(*r*)) in the magnetic core. The reference directions of the electric charge linkage (Γ) and Φ comply with the right-hand rule. L(*r*_0_) is used as the basis value to derive the normalized expression for L(*r*), and this is plotted in Fig. 4c. Magductance increases as |*r*| decreases, which indicates that the location is closer to the center of magnetic core. This tendency becomes stronger as frequency *f* increases. According to Ampere's law, the alternating MMF (F) is given by

(20) $$\begin{array}{c} F=H\left(r_{0}\right)l_{w}=\frac{l_{w}}{\mu }B\left(r_{0}\right)=\frac{l_{w}}{\mu }\frac{k\Phi }{2\pi r_{0}}\frac{I_{0}\left(kr_{0}\right)}{I_{1}\left(kr_{0}\right)} \end{array}$$

According to the law of the vector magnetic circuit [11,18,19], magnetic impedance Z-- is derived as follows:

(21) $$\begin{array}{ccc} Z\text{--} & = & \frac{F}{\Phi }=\frac{kl_{w}}{2\pi \mu r_{0}}\frac{I_{0}\left(kr_{0}\right)}{I_{1}\left(kr_{0}\right)}\\ & = & \frac{l_{w}}{\sqrt{2}\pi \mu \delta r_{0}}\frac{\mathrm{ber}\left(\alpha_{0}\right)+j\mathrm{bei}\left(\alpha_{0}\right)}{\mathrm{be}i^{\prime }\left(\alpha_{0}\right)-j\mathrm{be}r^{\prime }\left(\alpha_{0}\right)}=R+j\omega L \end{array}$$

Hence, the expression for reluctance R is given by

(22) $$\begin{array}{c} R=\frac{l_{w}}{\sqrt{2}\pi \mu \delta r_{0}}\frac{\mathrm{ber}\left(\alpha_{0}\right)\mathrm{be}i^{\prime }\left(\alpha_{0}\right)-\mathrm{bei}\left(\alpha_{0}\right)\mathrm{be}r^{\prime }\left(\alpha_{0}\right)}{{\left[\mathrm{be}i^{\prime }\left(\alpha_{0}\right)\right]}^{2}+{\left[\mathrm{be}r^{\prime }\left(\alpha_{0}\right)\right]}^{2}} \end{array}$$

The expression for magductance L is

(23) $$\begin{array}{c} L=\frac{l_{w}}{\sqrt{2}\pi \omega \mu \delta r_{0}}\frac{\mathrm{ber}\left(\alpha_{0}\right)\mathrm{be}r^{\prime }\left(\alpha_{0}\right)+\mathrm{bei}\left(\alpha_{0}\right)\mathrm{be}i^{\prime }\left(\alpha_{0}\right)}{{\left[\mathrm{be}i^{\prime }\left(\alpha_{0}\right)\right]}^{2}+{\left[\mathrm{be}r^{\prime }\left(\alpha_{0}\right)\right]}^{2}} \end{array}$$

Then, magnetic impedance angle *φ* is

(24) $$\begin{array}{c} \phi =\text{arctan}\frac{\omega L}{R} \end{array}$$

It should be noted that the values of reluctance and magductance can also be calculated by combining Poynting's vector [20] and the magnetoelectric power law [11,21], and the same results can be obtained as those obtained using (22), (23). The expressions for reluctance R_LF_ and magductance L_LF_ under a uniform magnetic flux density are as follows:

(25) $$\begin{array}{c} R_{\text{LF}}=\frac{l_{w}}{\mu \pi r_{0}^{2}} \end{array}$$

(26) $$\begin{array}{c} L_{\text{LF}}=\frac{\sigma l_{w}}{8\pi } \end{array}$$

The details of the derivation can be found in Appendix E. Combining Eq. 25 with (22), (26) with Eq. 23, the expressions of the normalized values for reluctance and magductance are given by

(27) $$\begin{array}{c} \frac{R}{R_{\text{LF}}}=\frac{\alpha_{0}}{2}\frac{\mathrm{ber}\left(\alpha_{0}\right)\mathrm{be}i^{\prime }\left(\alpha_{0}\right)-\mathrm{bei}\left(\alpha_{0}\right)\mathrm{be}r^{\prime }\left(\alpha_{0}\right)}{{\left[\mathrm{be}i^{\prime }\left(\alpha_{0}\right)\right]}^{2}+{\left[\mathrm{be}r^{\prime }\left(\alpha_{0}\right)\right]}^{2}} \end{array}$$

(28) $$\begin{array}{c} \frac{L}{L_{\text{LF}}}=\frac{4}{\alpha_{0}}\frac{\left[\mathrm{ber}\left(\alpha_{0}\right)\mathrm{be}r^{\prime }\left(\alpha_{0}\right)+\mathrm{bei}\left(\alpha_{0}\right)\mathrm{be}i^{\prime }\left(\alpha_{0}\right)\right]}{{\left[\mathrm{be}i^{\prime }\left(\alpha_{0}\right)\right]}^{2}+{\left[\mathrm{be}r^{\prime }\left(\alpha_{0}\right)\right]}^{2}} \end{array}$$

The results obtained using Eqs. 27, 28, and 24 are plotted in Fig. 4d–f, respectively. The magnetic flux skin effect virtually never occurs under the condition of *δ* > *r*_0_. As a result, reluctance and magductance vary negligibly, and the magnetic impedance angle fluctuates within 12°. However, *δ* decreases as the frequency increases. Consequently, reluctance increases owing to the decrease in the equivalent cross sectional area of magnetic flux, whereas magductance decreases owing to the decrease in the equivalent cross-sectional area of eddy current. The magnetic impedance angle gradually increases with the frequency.

### 2.3 Breathable magnetic core based on magnetic flux skin effect

Based on the analysis in 2.1, 2.2, it is elucidated that magductance contributes to the magnetic flux skin effect, consequently decreasing the utilization of the magnetic core. The equivalent magductance of the magnetic circuit must be minimized to eliminate the magnetic flux skin effect. There are two conventional methods for decreasing the equivalent magductance of the magnetic circuit [22,23]. The first is to change the electromagnetic properties of the magnetic material, such as decreasing conductivity. The second is to reduce the length of eddy current by dividing the magnetic circuit. For example, laminated sheets are typically used instead of solid electrical steel to form magnetic circuits for electrical machines. However, the mechanisms of the aforementioned strategies have not been revealed, even though they have been widely used to reduce eddy current loss [23].

**Fig. 5:**
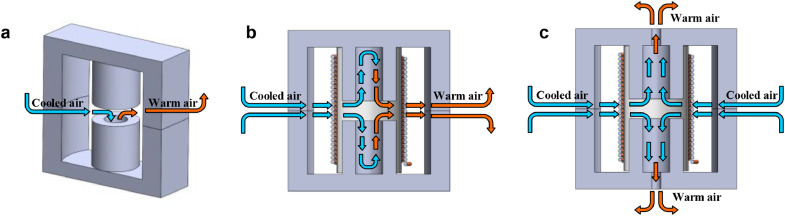
**Structure of a breathable magnetic core.** (a) Breathing process of EC type magnetic core. (b) Air circulation process for breathable magnetic core. Heat transfer is achieved when air passes through the hole structure in the center column in the magnetic core. (c) Air circulation in through-hole magnetic core, forming a complete loop.

The distribution curves of the normalized magnetic flux density and magductance in Fig. 4a,c show that magnetic flux is the minimum and magductance is the maximum at the center of the magnetic core under high-frequency field excitation. Therefore, based on the distribution of magductance in the magnetic core under different frequencies, the central part of the magnetic circuit is replaced by air or other high-resistivity materials to form a bracelet-shaped structure within the magnetic circuit, as shown in Fig. 5. Unlike the conventional method of inserting an air gap in series, the magnetic core design presented in this study incorporates an air hole in parallel with the magnetic circuit. This parallel configuration allows for the adjustment of the magnetic flux magnitude while modifying its distribution across the cross section of the magnetic core. When the magnetic core operates at high frequencies, this bracelet-like structure not only preserves the original magnetic flux but also effectively reduces the equivalent magductance, thereby improving the magnetic flux skin effect. According to the magnetoelectric power law [11,21], magductance corresponds to the active power of the magnetic circuit. Therefore, loss in the magnetic core can be decreased by reducing the equivalent magductance.

Additionally, the bracelet-shaped structure minimizes weight and cost and creates an internal channel within the magnetic core, thus enabling it to “breathe” and facilitating airflow. Therefore, we refer to the magnetic cores with the bracelet-shaped structure as “breathable magnetic cores.”

Fig. 5a shows the breathable magnetic core, by effectively increasing the surface area of the magnetic core, and thus the cooling efficiency of the magnetic core is improved. The air circulation through the magnetic core is similar a person breathing in and out. The bracelet-shaped structure of the magnetic core increases the heat dissipation area, which facilitates the circulation of cold air inside the core and improves the cooling effect, as shown in Fig. 5b. Furthermore, a continuous cooling circuit can be formed so that the hollow duct is extended to the two sides of magnetic core, as shown in Fig. 5c. This structure improves the effectiveness of cooling. However, it increases the equivalent reluctance of the magnetic circuit owing to the introduction of an additional air gap. This must be addressed.

Here, we describe the procedure for estimating the size of the hole in the magnetic core. First, the conductivity and the permeability of the magnetic core must be determined. The conductivity and permeability of the prototype magnetic core used in this study are 52 S/m and 4π × 10^−3^H/m, respectively. Next, the skin depth of the magnetic core is calculated based on the operating frequency of the magnetic core using Eq. 15. When the operating frequency is 20 kHz, the skin depth is calculated as 4.94 mm. Then, the corresponding curves are determined from Fig. 4 based on the ratio of *r*_0_ and *δ*. Finally, the size of the center hole of the magnetic core is determined using the corresponding curves. The diameter of the magnetic core is 30 mm, and the ratio of the radius to the skin depth is 3.036. This is close to the red curve shown in Figs. 4a,c. Note that the gradient of the red curve considerably decreases at approximately *r*/*r*_0_ = 0.5. Thus, this point should be used to determine the size of the magnetic core hole.

## 3 Experimental results

**Fig. 6:**
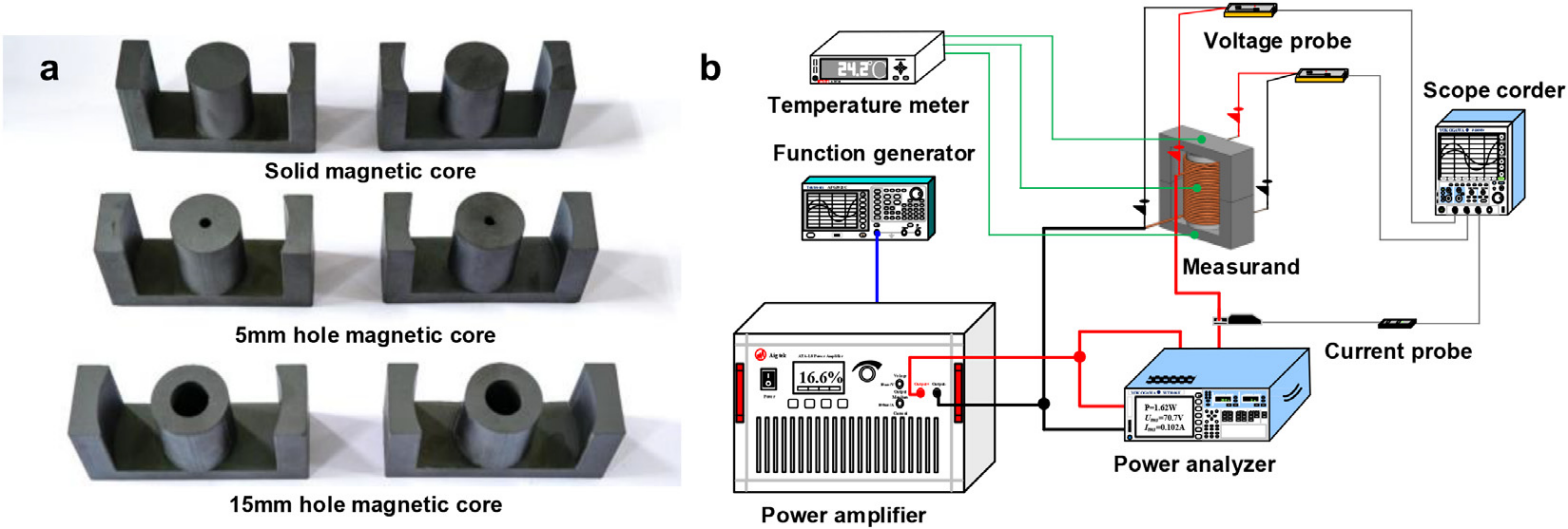
**Tested magnetic cores and the experimental platform.** (a) Three groups of magnetic cores, which are the solid magnetic core, the magnetic core with a 5 mm diameter hole, and the magnetic core with a 15 mm diameter hole. Two pieces of magnetic cores with same structure form a closed magnetic circuit. (b) Test platform for measuring MMF, magnetic flux, and active power of the magnetic core.

**Fig. 7:**
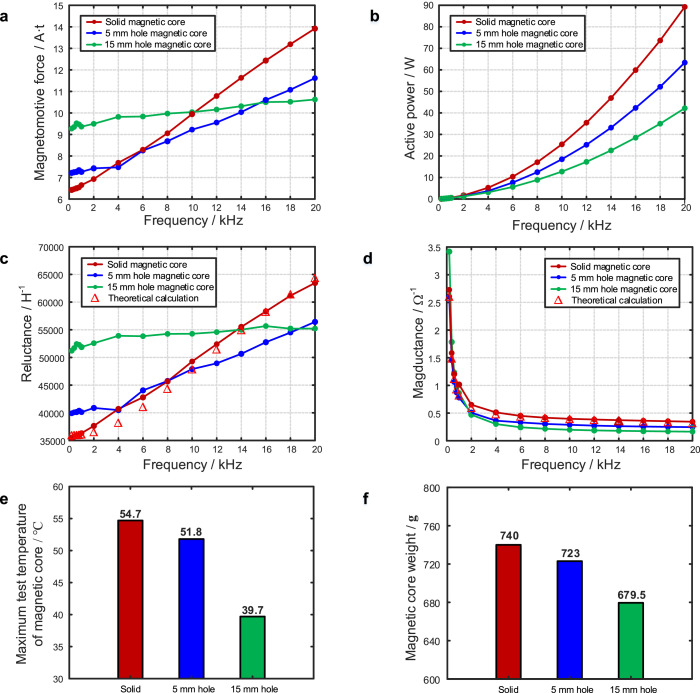
**Experimental results of magnetic circuit parameters for three kinds of magnetic cores.** (a) MMF of the three kinds of magnetic cores versus frequency under constant magnetic flux amplitude. (b) Active power of the three kinds of magnetic cores versus frequency, where the active power of the solid magnetic core is significantly higher than those of the magnetic cores with holes at high frequencies. (c) Reluctance of the three magnetic cores versus frequency. Reluctance increases with frequency owing to the magnetic flux skin effect. (d) Magductance of the three magnetic cores versus frequency. Magductance decreases as frequency increases because of the magnetic flux skin effect. (e) Maximum test temperatures for the magnetic cores during the experiment, the magnetic core with the 15 mm hole exhibits the lowest temperature, whereas the solid core demonstrates the highest temperature. (f) Weights of solid magnetic core and magnetic cores with the 5 mm and 15 mm holes are 740 g, 723 g, and 679.5 g, respectively.

To verify the theoretical analysis, we fabricate three sets of magnetic cores with different hole diameters, as shown in Fig. 6a. To ensure that the eddy current losses in the magnetic core are significantly greater than the hysteresis losses, we use HP3 material from NCD for fabricating the cores. We set up an experimental platform, as shown in Fig. 6b. We change the frequency of magnetic flux from 20 Hz to 20 kHz while maintaining the magnitude of magnetic flux at approximately 0.00018 Wb. The MMF, magnetic flux, and active power are monitored and recorded. The experimental results are shown in Fig. 7a–d. The detailed experimental parameters, instrument type, and test procedures are described in Appendix B.

The equivalent reluctance of the magnetic circuit increases at lower frequencies because of the hole in the magnetic core. Hence, Fig. 7a shows that at frequencies below 10 kHz, a larger MMF is required to generate the same magnetic flux in the magnetic core containing a hole with a diameter of 15 mm than that in the solid magnetic core. However, the reluctance of the solid magnetic core increases at higher frequencies owing to the magnetic flux skin effect and the increase in magnetic reactance. Therefore, the MMF required to generate the same magnetic flux for the magnetic core with the 15 mm hole is lower than that required for the solid magnetic core at frequencies above 10 kHz (Fig. 7a). Additionally, the active power of the magnetic cores with holes considerably decreases in the high-frequency range of magnetic flux (Fig. 7b). This implies that these magnetic cores have a lower power loss. Therefore, the temperature rise of magnetic core reduces, and efficiency is improved.

The equivalent reluctance and magductance of the magnetic circuit are obtained at various frequencies using the vector magnetic circuit theory or magnetoelectric power law [11,21], as shown in Figs. 7c and d. The hole at the center causes the equivalent reluctance of magnetic core to become insensitive to frequency changes. Instead, the solid magnetic core experiences a strong magnetic flux skin effect, which causes the equivalent reluctance to increase significantly with the frequency (Fig. 7c). It should be noted that the magductance of all magnetic cores decreases as the frequency increases, regardless of whether they have holes or not. This is because the eddy current skin effect in the magnetic core reduces magductance (Fig. 7d). However, the equivalent magductance of the breathable magnetic core is relatively lower than that of the solid magnetic core. The theoretical analysis results obtained using (22), (23) are in good agreement with the test results, thereby verifying the accuracy of the theoretical analysis. The maximum test temperatures at the same positions, namely, the center columns for the three sets of magnetic cores, are monitored in real time using thermocouples during the testing, as shown in Fig. 7e. The solid core exhibits the highest temperature, followed by the cores with the 5 mm and 15 mm holes. This verifies the previous theoretical analysis. According to the measurement results obtained using the weighting meter, as shown in Fig. 7f, the weight of the solid magnetic cores and the cores with the 5 mm and 15 mm holes are 740 g, 723 g, and 679.5 g, respectively. A maximum reduction of 8.17% in weight is achieved using the 15 mm hole.

## 4 Conclusion

We have established the inherent relationship between the magductance of a magnetic core and the magnetic flux skin effect and revealed the physical nature of the magnetic flux skin effect for the first time. On this basis, a novel breathable magnetic core with a hole at the center is proposed based on the magnetic flux skin effect to improve the performance of high-frequency electromagnetic components. Moreover, the general expressions of reluctance and magductance at different frequencies are presented and verified by testing the solid magnetic core.

With the rapid development of power electronics, particularly the widespread application of wide bandgap power electronic devices, the frequency of the magnetic flux that passes through magnetic cores has increased, and the magnetic flux skin effect has become stronger. The proposed methodology for analysis of the magnetic flux skin effect can be applied to magnetic cores with different shapes. Therefore, this fundamental study sheds new light on the optimal design of magnetic components to improve their efficiency, cost, size, etc.

## Declaration of competing interest

The authors declare that they have no conflicts of interest in this work.

## Figures and Tables

**Table A1:** **Details of experiment for the magnetic flux skin effect**.

Parameters/Instruments	Value/type
Iron core material	DT4C
Insulating paper	DMD 0.2 mm
Adhesive tape	Polyimide film 0.05 mm
Primary-side winding	ZR-BVR4.0^2^
Number of turns on primary side	150
Primary-side DC resistance	0.29 Ω
Secondary-side winding	Enameled copper wire 0.47 mm
Number of turns on secondary side	50
Secondary-side DC resistance	1.33 Ω
Magnetic powder	Fe_3_O_4_ 400 mesh
Testing sieve	325 mesh 100 × 45 mm
Petri dish	Polystyrene 90 × 15 mm
Programmable AC power supply	ITECH IT7627
Power analyzer	YOKOGAWA WT1806E
Temperature meter	Anbai AT4617

**Table:** **Table B1 Details of the breathable magnetic core experiment**.

Parameters/Instruments	Value/type
Magnetic core model	EC90
Magnetic core material	NCD HP3
Magnetic core conductivity	52 S/m
Magnetic core permeability	4π × 10^−3^H/m
Equivalent magnetic circuit length	0.32 m
Saturation flux density	400 mT
Adhesive tape	Polyimide film 0.05 mm
Primary-side winding	Litz wire 0.1 mm/100
Number of turns on primary side	25
Primary-side DC resistance	0.0717 Ω
Secondary-side winding	Enameled copper wire 0.47 mm
Number of turns on secondary side	25
Secondary-side DC resistance	0.275 Ω
Magnetic flux amplitude	0.00018 Wb
Function generator	Tektronix AFG3021C
Power amplifier	Aigtek ATA-L8
Power analyzer	YOKOGAWA WT1806E
Scope recorder	YOKOGAWA DLM3024
Temperature meter	Anbai AT4617
Voltage probe	Sapphire Instrument SI-9002
Current probe	CYBERTEK HCP8050

**Fig. B1:**
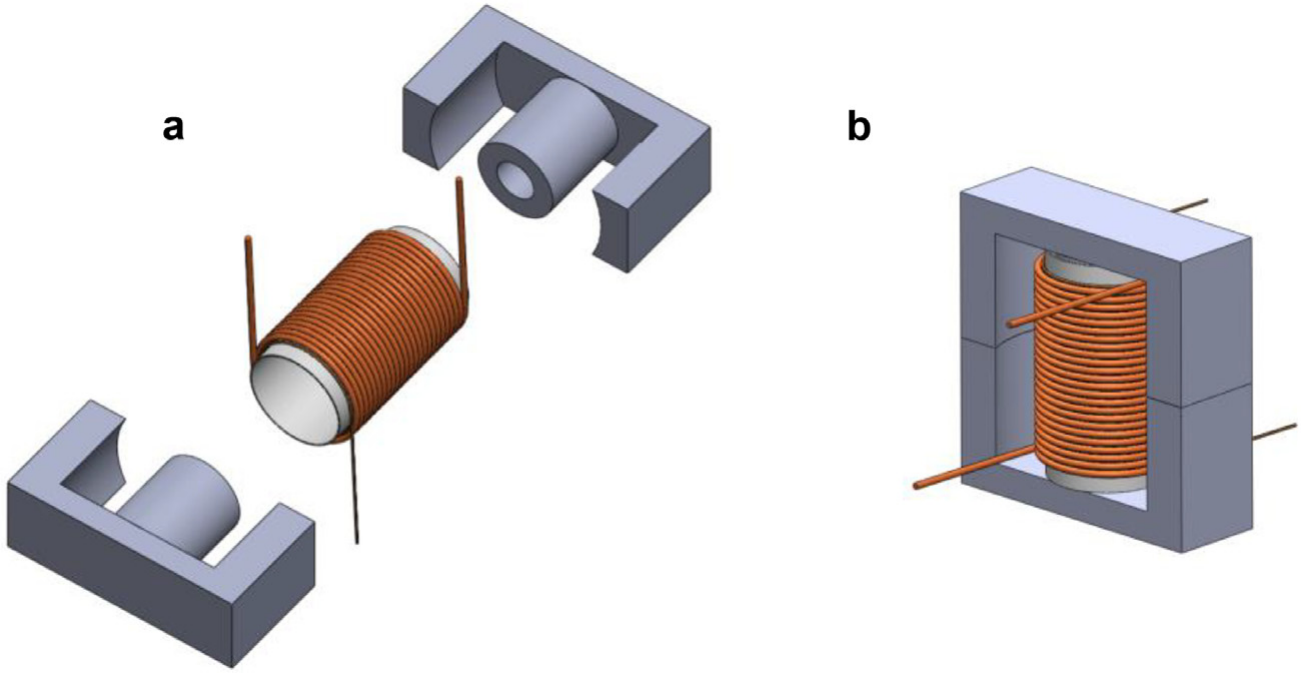
**Formation of the closed magnetic circuit.** (a) Replacement of the magnetic core. Different magnetic cores are tested with the same primary-side and secondary-side windings. (b) Two identical magnetic cores form a closed magnetic circuit.

## Appendix A Magnetic flux skin effect experiment

The procedure of experiment is shown in Fig. A1. First, we use a sieve to evenly spread a thin layer of the magnetic powder on a Petri dish (Fig. A1a) and check whether the distribution of the magnetic powder is even under light (Fig. A1b). If the magnetic material is not dispersed uniformly, it is spread again. Next, the Petri dish is placed into the air gap position in the electrical steel, and it is ensured that the Petri dish is at the center of the cross section (Fig. A1c). To maintain a constant MMF, the output voltage of the programmable AC power supply is adjusted to be the testing frequency until the RMS value of current in the primary winding is 30 A. The power supply is turned off after supplying current for approximately 20 s. Then, the Petri dish is removed by maintaining the same direction, and images are obtained using a camera for recording the distribution of the magnetic powder. The test at one frequency is completed in this manner. The magnetic powder distribution is measured three times for each frequency. The experimental results are considered to be reliable when the distribution of the magnetic powder in these three measurements is consistent. Temperature increases rapidly owing to large eddy current, which affects the parameters of the magnetic circuit for the solid electrical steel. Therefore, the temperature of the solid electrical steel is monitored and maintained below 30 °C to remove the effect of temperature change [24]. The experimental details are listed in Table A1.

We calculate the MMF and magnetic flux of the solid electrical steel at different frequencies using the measured data obtained from the power analyzer. The primary-side current is used to solve the MMF according to Ampere's law, and the secondary-side back electromotive force (EMF) is used to solve the magnetic flux using Faraday's law [14].

## Appendix B Experiment with a breathable magnetic core

The instruments used in this experiment and the relevant experimental parameters are shown in Fig. 6b and Table B1. The function generator produces an adjustable-frequency sine wave, and a power amplifier is used to supply power to the primary side of the magnetic core. Thus, alternating magnetic flux is produced in the magnetic circuit. The power analyzer records the values of current on the primary side, EMF values on the secondary side, and active power. The scope recorder records the waveforms of primary-side current and secondary-side EMF using a current probe and voltage probe, respectively. Each set of the magnetic core test is conducted at the same room temperature (approximately 25 °C), and the highest temperature in the magnetic core center column is monitored in real time. The wiring of the windings is unchanged, and only the magnetic core is replaced by following the procedure shown in Fig. B1a. Thus, the winding parameters of various magnetic cores are ensured to be consistent throughout the experiment. A closed magnetic circuit is created using two identical magnetic cores, as shown in Fig. B1b.

The MMF and magnetic flux are calculated in the same manner as that in the prior experiments. The active power of the magnetic core is measured by the power analyzer. In addition, the reluctance and magductance of the magnetic core are calculated using the vector magnetic circuit theory. (22), (23) are used to calculate the theoretical values of the reluctance and magductance of the magnetic cores, and the relevant parameters are shown in Table B1. Eddy current loss, hysteresis loss, and anomalous loss are three types of magnetic circuit loss [25], and they affect the calculation of magductance. As excitation frequency increases, eddy current loss increases considerably more than hysteresis loss. Anomalous loss is equivalent to the reduced conductivity of the magnetic core [26]. The conductivity is reduced to 30 S/m in the theoretical calculation of magductance.

## Appendix C Derivation of Bessel equation for the magnetic core

Consider a cylindrical magnetic core with radius *r*_0_, as shown in Fig. 3a. The magnetic flux density (*B*) and magnetic field intensity (*H*) have only axial components. *B* is not uniform in the magnetic core at high frequencies, and it depends on the distance of the testing point from the center of the magnetic core (*r*), i.e., *B*(*r*). The cross-sectional area of a shell with radius *r* and thickness d*r* is *S* = 2π*r*d*r*. The magnetic flux (Φ(*r*)) that flows through the shell is

(C.1) $$\Phi \left(r\right)=\int_{0}^{r}BdS=\int_{0}^{r}B2\pi rdr$$

From Maxwell's Equations (only considering the magnitude), we obtain

(C.2) $$\oint_{C}E\cdot dl=E2\pi r=\left|\frac{d\Phi \left(r\right)}{dt}\right|=\frac{d\left(\int_{0}^{r}B2\pi rdr\right)}{dt}$$

where *E* denotes the electric field strength. This gives

(C.3) $$Er=\frac{d\left(\int_{0}^{r}Brdr\right)}{dt}$$

Differentiating *r* on both sides of this equation, the following equation is derived:

(C.4) $$\frac{dE}{dr}+\frac{E}{r}-\frac{dB}{dt}=0$$

The eddy current (*I*) that flows through the cross-sectional area of the shell with length *l* and thickness d*r* is

(C.5) I=Jldr=σEldr

where *J* denotes the current density. According to Ampere's Law [6], the eddy current (*I*) induced by the time-varying magnetic flux in the shell is

(C.6) I=Hl

This yields the magnetic field intensity (*H*) as follows:

(C.7) H=σEdr

Then, the magnetic flux density on the outer surface of the shell (*B*_0_) in terms of the magnetic flux density on its inner surface (*B*) is given by

(C.8) $$B_{0}=B+\frac{\partial B}{\partial r}dr$$

The resulting magnetic field intensity (*H*_0_) on the outer surface is

(C.9) $$H_{0}=\frac{1}{\mu }\left(B+\frac{\partial B}{\partial r}dr\right)$$

Therefore, the change in the magnetic field intensity from the inner to the outer surface of shell is given by

(C.10) $$H=\frac{1}{\mu }\frac{\partial B}{\partial r}dr$$

As *B* only has the axial component, equating Eq. C.7 and Eq. C.10, we obtain:

(C.11) $$E\sigma \mu =\frac{dB}{dr}$$

Differentiating *r* on both sides of this equation, we obtain

(C.12) $$\frac{dE}{dr}\sigma \mu =\frac{d^{2}B}{dr^{2}}$$

Substituting Eq. C.11 and Eq. C.12 into Eq. C.4 yields

(C.13) $$\frac{d^{2}B}{dr^{2}}+\frac{1}{r}\frac{dB}{dr}-\sigma \mu \frac{dB}{dt}=0$$

The phasor description is used for the sinusoidal magnetic flux that flows through the magnetic core. This gives

(C.14) $$\frac{dB}{dt}=j\omega B$$

where *ω* denotes the angular frequency of *B*. By transferring Eq. C.13 from the time domain to the frequency domain, the following equation can be obtained:

(C.15) $$\frac{d^{2}B}{dr^{2}}+\frac{1}{r}\frac{dB}{dr}-j\omega \sigma \mu B=0$$

Differentiating *r* on both sides of Eq. C.4, the following relationship is obtained:

(C.16) $$\frac{d^{2}E}{dr^{2}}+\frac{1}{r}\frac{dE}{dr}-\frac{E}{r^{2}}-\frac{d^{2}B}{dtdr}=0$$

Differentiating both sides of Eq. C.11 with respect to *t* gives

(C.17) $$\sigma \mu \frac{dE}{dt}=\frac{d^{2}B}{drdt}$$

Substituting Eq. C.17 into Eq. C.16 yields

(C.18) $$\frac{d^{2}E}{dr^{2}}+\frac{1}{r}\frac{dE}{dr}-\frac{E}{r^{2}}-\sigma \mu \frac{dE}{dt}=0$$

Combining Eq. C.14 and Eq. C.18, we obtain the Bessel equation in the phasor form as

(C.19) $$\frac{d^{2}E}{dr^{2}}+\frac{1}{r}\frac{dE}{dr}-\frac{E}{r^{2}}-j\omega \sigma \mu E=0$$

## Appendix D Derivation of magductance distribution for a cylindrical magnetic core

According to Eq. 18, the magnetic flux enclosed by a cylindrical shell with radius *r* is

(D.1) $$\Phi \left(r\right)=\int_{0}^{r}B\left(r\right)2\pi rdr=\frac{\Phi I_{1}\left(kr\right)}{I_{1}\left(kr_{0}\right)}\left(\frac{r}{r_{0}}\right)$$

According to the properties of the Bessel function, the following equation is obtained:

(D.2) $$\int xI_{0}\left(x\right)dx=xI_{1}\left(x\right)$$

Hence,

(D.3) $$\frac{d\Phi \left(r\right)}{dr}=k\Phi \frac{I_{0}\left(kr\right)}{I_{1}\left(kr_{0}\right)}\left(\frac{r}{r_{0}}\right)$$

The ratio of the magnetic flux that flows through a cylindrical shell with the radius ranging from *r* to *r* + d*r* is given by

(D.4) $$\frac{d\Phi \left(r\right)}{\Phi }=k\frac{I_{0}\left(kr\right)}{I_{1}\left(kr_{0}\right)}\left(\frac{r}{r_{0}}\right)dr$$

As shown in Fig. 3a, current is generated between the cylindrical shell with radius *r* and the magnetic core surface with radius *r*_0_ by the magnetic flux enclosed inside the shell.

(D.5) $$I\left(r\right)=\underset{S}{\;\int \int \;}J\left(r\right)dr=\int_{0}^{l_{w}}\int_{r}^{r_{0}}\sigma E\left(r\right)drdz=\frac{j\omega \Phi }{2\pi kr_{0}}\frac{l_{w}\sigma }{I_{1}\left(kr_{0}\right)}\int_{r}^{r_{0}}I_{1}\left(kr\right)d\left(kr\right)$$

The current linkage (Θ) indicates the current generated per unit magnetic flux (Φ) in the *N*-turn magductance component. Here, Θ(*r*) denotes the current linkage generated by magnetic flux Φ(*r*) in the magnetic core. According to Eq. D.2, Eq. D.4, and Eq. D.5, Θ(*r*) is expressed as

(D.6) $$\begin{array}{ccc} \Theta \left(r\right) & = & \int_{0}^{r}I\left(r\right)\frac{d\Phi \left(r\right)}{\Phi }\\ & = & \frac{j\omega \Phi l_{w}\sigma }{2\pi {\left(kr_{0}\right)}^{2}I_{1}^{2}\left(kr_{0}\right)}\int_{0}^{kr}\left[I_{0}\left(kr_{0}\right)-I_{0}\left(kr\right)\right]I_{0}\left(kr\right)\left(kr\right)d\left(kr\right)\\ & = & \frac{j\omega \Phi l_{w}\sigma \left(kr\right)}{2\pi {\left(kr_{0}\right)}^{2}I_{1}^{2}\left(kr_{0}\right)}\left\{I_{0}\left(kr_{0}\right)I_{1}\left(kr\right)-\frac{kr}{2}\left[I_{0}^{2}\left(kr\right)-I_{1}^{2}\left(kr\right)\right]\right\} \end{array}$$

The charge linkage (Γ) indicates the number of free charges generated per unit magnetic flux Φ in the *N*-turn magductance component. Here, Γ(*r*) denotes the charge linkage generated by magnetic flux Φ(*r*) in the magnetic core. Hence, the distributed charge linkage (Γ(*r*)) is expressed as

(D.7) $$\begin{array}{ccc} \Gamma \left(r\right) & = & \int \Theta \left(r\right)dt\\ & = & \frac{\Phi l_{w}\sigma \left(kr\right)}{2\pi {\left(kr_{0}\right)}^{2}I_{1}^{2}\left(kr_{0}\right)}\left\{I_{0}\left(kr_{0}\right)I_{1}\left(kr\right)-\frac{kr}{2}\left[I_{0}^{2}\left(kr\right)-I_{1}^{2}\left(kr\right)\right]\right\} \end{array}$$

Magductance is defined as the ratio of the electric charge linkage to the linked magnetic flux of the magnetic circuit. Hence, distributed magductance (L(*r*)) is expressed as

(D.8) $$\begin{array}{ccc} L\left(r\right) & = & \frac{\Gamma \left(r\right)}{\Phi \left(r\right)}\\ & = & \frac{l_{w}\sigma }{2\pi \left(kr_{0}\right)I_{1}\left(kr_{0}\right)I_{1}\left(\mathrm{kr}\right)}\left\{I_{0}\left(kr_{0}\right)I_{1}\left(\mathrm{kr}\right)-\frac{\mathrm{kr}}{2}\left[I_{0}^{2}\left(\mathrm{kr}\right)-I_{1}^{2}\left(\mathrm{kr}\right)\right]\right\}\\ & = & \frac{l_{w}\sigma }{2\pi }\left\{\frac{1}{kr_{0}}\frac{I_{0}\left(kr_{0}\right)}{I_{1}\left(kr_{0}\right)}-\frac{1}{2}\left(\frac{r}{r_{0}}\right)\left[\frac{I_{0}^{2}\left(\mathrm{kr}\right)}{I_{1}\left(kr_{0}\right)I_{1}\left(\mathrm{kr}\right)}-\frac{I_{1}\left(\mathrm{kr}\right)}{I_{1}\left(kr_{0}\right)}\right]\right\} \end{array}$$

## Appendix E Derivation of expressions for reluctance and magductance under uniform magnetic flux

At low frequencies, the magnetic flux density (*B*) in the magnetic core is uniform. Therefore, the magnetic flux density of the entire magnetic core is

(E.1) $$B\left(r_{0}\right)=\frac{\Phi }{\pi r_{0}^{2}}$$

where Φ denotes the total magnetic flux that flows through the magnetic core. Therefore, the magnetic flux density for a part of the magnetic core with radius *r* is

(E.2) $$B\left(r\right)=\frac{\Phi \left(r\right)}{\pi r^{2}}$$

As *B*(*r*) =*B* (*r*_0_), the following relationship is obtained:

(E.3) $$\Phi \left(r\right)=\Phi {\left(\frac{r}{r_{0}}\right)}^{2}$$

From Maxwell's Equations, the electric field intensity (*E*(r)) inside the magnetic core with radius *r* is given by

(E.4) $$E\left(r\right)=\frac{\frac{\Phi \left(r\right)}{dt}}{2\pi r}=\frac{j\omega \Phi r}{2\pi r_{0}^{2}}$$

From Poynting's vector, we can calculate active power *P* as follows:

(E.5) $$P=\underset{V}{\;\int \int \int \;}\left|\sigma E{\left(r\right)}^{2}\right|dV=\sigma l_{w}\frac{\omega^{2}\Phi^{2}}{2\pi r_{0}^{4}}\int_{0}^{r_{0}}r^{3}dr=\sigma l_{w}\frac{\omega^{2}\Phi^{2}}{8\pi }$$

We use the magnetoelectric power law [11,21] expressed as

(E.6) $$\begin{array}{c} P=\omega \left(\omega L\right)\Phi^{2} \end{array}$$

By equating Eq. E.5 and Eq. E.6, the magductance value under uniform magnetic flux is obtained as

(E.7) $$\begin{array}{c} \text{L}=\frac{\sigma l_{w}}{8\pi } \end{array}$$

Further, magnetic field energy *W_m_* is obtained using Poynting's vector, as follows:

(E.8) $$W_{m}=\frac{1}{2}\underset{V}{\;\int \int \int \;}\left|\frac{B{\left(r\right)}^{2}}{\mu }\right|dV=\frac{l_{w}\Phi^{2}}{\mu \pi r_{0}^{4}}\int_{0}^{r_{0}}rdr=\frac{l_{w}\Phi^{2}}{2\mu \pi r_{0}^{2}}$$

According to the electric circuit theory, the expression for *W_m_* is obtained as

(E.9) $$\begin{array}{c} W_{m}=\frac{1}{2}LI^{2}=\frac{1}{2}\frac{N^{2}I^{2}}{R}=\frac{1}{2}\frac{F^{2}}{R}=\frac{1}{2}R\Phi^{2} \end{array}$$

By equating Eq. E.8 and Eq. E.9, the reluctance value under uniform magnetic flux is obtained as

(E.10) $$\begin{array}{c} R_{\text{LF}}=\frac{l_{w}}{\mu \pi r_{0}^{2}} \end{array}$$
